# MRI-based anatomical and biomechanical characterization of the human masticatory muscles

**DOI:** 10.3389/fbioe.2026.1888163

**Published:** 2026-09-14

**Authors:** Thomas Zöchmeister, Lukas Berger-Maul, Martin Tik, Erik Kornfellner

**Affiliations:** 1 UAS Campus Vienna, Vienna, Austria; 2 Medical University of Vienna, Vienna, Austria; 3 UAS Technikum Vienna, Vienna, Austria

**Keywords:** biomechanical phenomena, computer-assisted, image processing, magnetic resonance imaging, masticatory muscles, sex characteristics

## Abstract

Biomechanical models of the masticatory muscles quantify the complex force and movement patterns of the masticatory system and are essential for evidence-based diagnosis, treatment planning, and prosthetic care in the craniomandibular region. This study provides comprehensive, sex-specific parameters for assessing the morphology of the masticatory muscles in young adult males and females, based on high-resolution magnetic resonance imaging (MRI) images and semi-automatic three-dimensional (3D) segmentation. Parameters such as volume, length, anatomical cross-sectional area and surface area of the masseter muscle, pterygoid muscle and temporalis muscle are evaluated bilaterally. Their force application points on the skull and mandible are determined using a new center-of-mass-based segmentation method for biomechanical model interpretation. Statistical analysis indicates that 66 of 68 parameters show significant sex-specific differences, mostly with large effect sizes: male participants generally showed higher volumes, lengths, and cross-sectional areas, except for two cross-sectional areas of the superficialis masseter muscle, resulting in higher surface-to-volume ratios in women. Although minor asymmetrical effects are observed, no significant lateral differences are found within each sex, suggesting bilateral symmetry of the masticatory muscles in the study cohort. Comparing volume data with existing literature shows that this study’s data generally exceeds historical cadaver measurements but is consistent with recent digital studies, underscoring the influence of age, methodology, and sex-specific differences on the interpretation of morphometric muscle parameters. Overall, this research provides a valuable foundation for the morphometric characterization and sex-specific biomechanical modeling of the masticatory musculature, while also highlighting the limitations of small, unbalanced, and mixed-sex reference groups.

## 1 Introduction

The specific forces generated by the masticatory muscles are essential for chewing, occlusion, and stress production in the temporomandibular joint (TMJ) (Hannam et al., 2008; Koolstra et al., 1988). Additionally, these muscles are involved in mastication, swallowing, and speaking (Sagl et al., 2019), which is why they play a central role in temporomandibular disorders (TMDs), bruxism, and jaw-related clinical treatments (Baron and Debussy, 1979; Hannam et al., 2008). The multipennate and independent structure of the masticatory muscles produces complex force-vector directions and supports a dynamic muscle model with six degrees of freedom (Koolstra and Van Eijden, 1997). In this model, the volume and maximum muscle fiber length are key factors in evaluating physiological cross-sectional area (PCSA) and maximum muscle force (*F*) (Gans and Bock, 1965; Baron and Debussy, 1979; Weijs and Hillen, 1985). Additionally, muscle parameters such as volume, length and cross-sectional area (CSA) serve as input data for biomechanical models and bite-force models, which, combined with muscle fiber length, simulate muscle tensions, joint forces, and jaw movements (Gans and Bock, 1965; Brand et al., 1986; Xu et al., 1994; Peck et al., 2000; Heins et al., 2025). For the quantitative assessment of these morphological parameters, *in vivo* imaging techniques like computed tomography (CT), MRI, and ultrasound are used (National Research Council, 1996; Heemskerk and Damon, 2007; Brink et al., 2025), enabling sex-specific and patient-specific biomechanical models and personalized clinical treatments, such as prosthetic joint replacements and mandibular reconstruction planning (Ackland et al., 2017; Sagl et al., 2019; Heins et al., 2025; Brink et al., 2025). To avoid misinterpretations of biomechanical models and ensure their clinical relevance, factors such as ethnicity, sex, age, and the sample size of studied cohorts should be considered (Newton et al., 1993; Palinkas et al., 2010; Tamura and Shiga, 2014; Merema et al., 2022; Yılmaz et al., 2023).

The current state of research on biomechanical models of the masticatory muscles indicates systematic deficits due to their greater complexity compared to established musculoskeletal models in other regions of the body, such as the knee, hip, or leg (Koolstra, 2002). Some masticatory muscles have irregular shapes, are pennate, and show large attachment areas to the skull and mandible (Koolstra and Van Eijden, 1992; 1996). Consequently, modern studies often rely on classic, established parameters, such as Baron and Debussy (1979), Van Eijden et al. (1997), Weijs and Hillen (1984). These data are often based on cadaver studies with small sample sizes and high average ages, limiting their comparability with young adult populations (Newton et al., 1993). Established literature on imaging characterization of the masticatory muscles mostly examines mixed cohorts without stratification by sex (Lin et al., 2025; Ng H. et al., 2009). However, recent MRI-based and predictive approaches further demonstrate the relevance of quantitative masticatory muscle parameters for clinical and biomechanical applications, highlighting the need for more detailed, patient- and sex-specific reference data (Jeon et al., 2024; Brink et al., 2025; Heins et al., 2025). Despite this relevance, separate data on male and female muscle parameters such as length, anatomical cross-sectional area, surface area, and surface-to-volume ratio are rarely investigated in a single study. Moreover, bilateral comparisons of healthy male and female muscle parameters, as well as sex-specific characterization and comparisons of these parameters, are mostly lacking. These gaps in the literature may directly impact the validity and clinical relevance of biomechanical models. If ethnicity, sex, age, and sample size are not properly considered, there is a higher risk of misinterpreting biomechanical models, which may lead to incorrect clinical treatments and therapies for patients.

The aim of this study was to use high-resolution MRI datasets to compare sex-specific morphometric parameters of the main masticatory muscles in young adult participants. In addition, bilateral differences were analysed, and the measured parameters were compared with previously published data to provide a basis for future biomechanical modelling of the masticatory system.

## 2 Materials and methods

### 2.1 Imaging data

The imaging data were collected at the Medical University of Vienna as part of earlier research projects. Therefore, the sample size was determined by the number of available eligible MRI datasets rather than by a formal pre-study sample size calculation. All studies were conducted in accordance with institutional guidelines and were approved by the ethics committee of the Medical University of Vienna (Number 1309/2018), and informed consent was obtained. To analyse sex differences in the morphology of masticatory muscles, the datasets were divided by sex into groups of medically unremarkable female and male subjects. The female cohort consisted of 15 subjects with a median age of 23 years (interquartile range (IQR): 20–26; range: 18–39), a median height of 170 cm (IQR: 161.5–170; range: 160–180), and a median body weight of 60 kg (IQR: 60–70; range: 50–80). The male group comprised 7 subjects with a median age of 22 years (IQR: 21.5–26; range: 19–29), a median height of 180 cm (IQR: 175–180; range: 170–180), and a median body weight of 80 kg (IQR: 80–80; range: 70–90). Body mass index (BMI) was not included as a control variable because body weight and height were available only as rounded values, limiting reliable BMI calculation or adjustment. The data were collected between November 7 2022 and 12 December 2023. All scans were performed using the Siemens Prisma Fit 3-Tesla (3-T) scanner (Siemens Healthineers AG, Munich, Germany), using the software version syngo MR E11, with a Body-Transmit Coil in head-first supine position. Sequence parameters included repetition time (*T*_R_) = 2100 ms, echo time (*T*_E_) = 3.67 ms, inversion time (*T*_I_) = 900 ms, flip angle = 8°, number of averages = 1, and imaging nucleus = ^1^H. The spatial resolution consisted of a 1 mm slice thickness with a pixel size of 1.0156 × 1.0156 mm (256 × 224 pixels per slide).

### 2.2 Image pre-processing

To prepare the MRI datasets in digital imaging and communications in medicine (DICOM) format, the open-source medical imaging computing program 3D Slicer (Version 5.10.0, URL www.slicer.org) was used for visualization, segmentation, and measurement of the masticatory muscles (Fedorov et al., 2012). Based on the literature, four main muscle groups involved in mastication for biomechanical modeling of jaw mechanics were initially defined: M. masseter (MM) M. pterygoideus medialis (MPM), M. pterygoideus lateralis (MPL), and M. temporalis (MT). The right and left muscles were segmented in both males and female participants to identify asymmetrical and sex-related differences. All segmentations and quantitative measurements were performed by a single investigator trained in anatomy and experienced in the processing and analysis of three-dimensional medical imaging data. The same standardized procedures were applied throughout the study.

The anatomical boundaries of the masticatory muscles are often difficult to identify in MRI data. Therefore, automatic segmentation methods are prone to errors and require extensive manual post-processing. Manual segmentation methods are precise but demand significant time resources and are highly user-dependent. A semi-automatic method using the image computing platform 3D Slicer was chosen as a compromise between accuracy and speed for segmenting the defined masticatory muscles. The semi-automatic approach in 3D Slicer involves manual pre-processing, automated processing of manually entered data, and final manual post-processing. The muscle segmentation process in this study was performed in the following order: (1) MM, (2) MPM, (3) MPL, and (4) ML. After segmenting the four main masticatory muscles, the M. masseter was divided into its two parts (M. masseter superficialis (MMS) and M. masseter profunda (MMP)) to obtain more precise landmark measurements and facilitate better comparison with existing literature, such as Van Eijden et al. (1997).

### 2.3 Segmentation method

The segmentation method used in this study on the imaging computing platform 3D Slicer is called “Grow from Seeds.” This module requires a manually pre-processed muscle layer (seed layer) and background layers, which are distinguished by the Slicer. A seed segmentation layer and a background layer were created for parts of the MM, as well as the medial and lateral pterygoid muscles and the temporal muscle. Initially, all regions corresponding to muscle tissue were manually marked in the selected muscle layer, while in the background layer, all tissues surrounding the muscle were segmented. Since accurate, manually pre-processed segmentation data results in better segmentation outcomes and reduces post-processing of calculation artifacts, at least 15 slices per anatomical plane (axial, sagittal, coronal) were manually marked for both the seed and background layers within the imaging platform. The algorithm of the imaging computing platform expanded from the seed voxels and was constrained by the intensity gradients of the marked voxels in the background layer. After calculating the muscle segments in the 3D Slicer software, areas with limited visual distinction between muscle and background, such as regions with low contrast due to bony structures, tendinous structures, or air cavities, were manually post-processed within the imaging computing platform to ensure accurate anatomical muscle boundaries. Following post-processing, a smoothing function was applied in 3D Slicer with a low-resolution factor of 3 × 3 × 3 voxels to reduce small surface irregularities and preserve anatomical structures, including origins and insertions (Figure 1).

**FIGURE 1:**
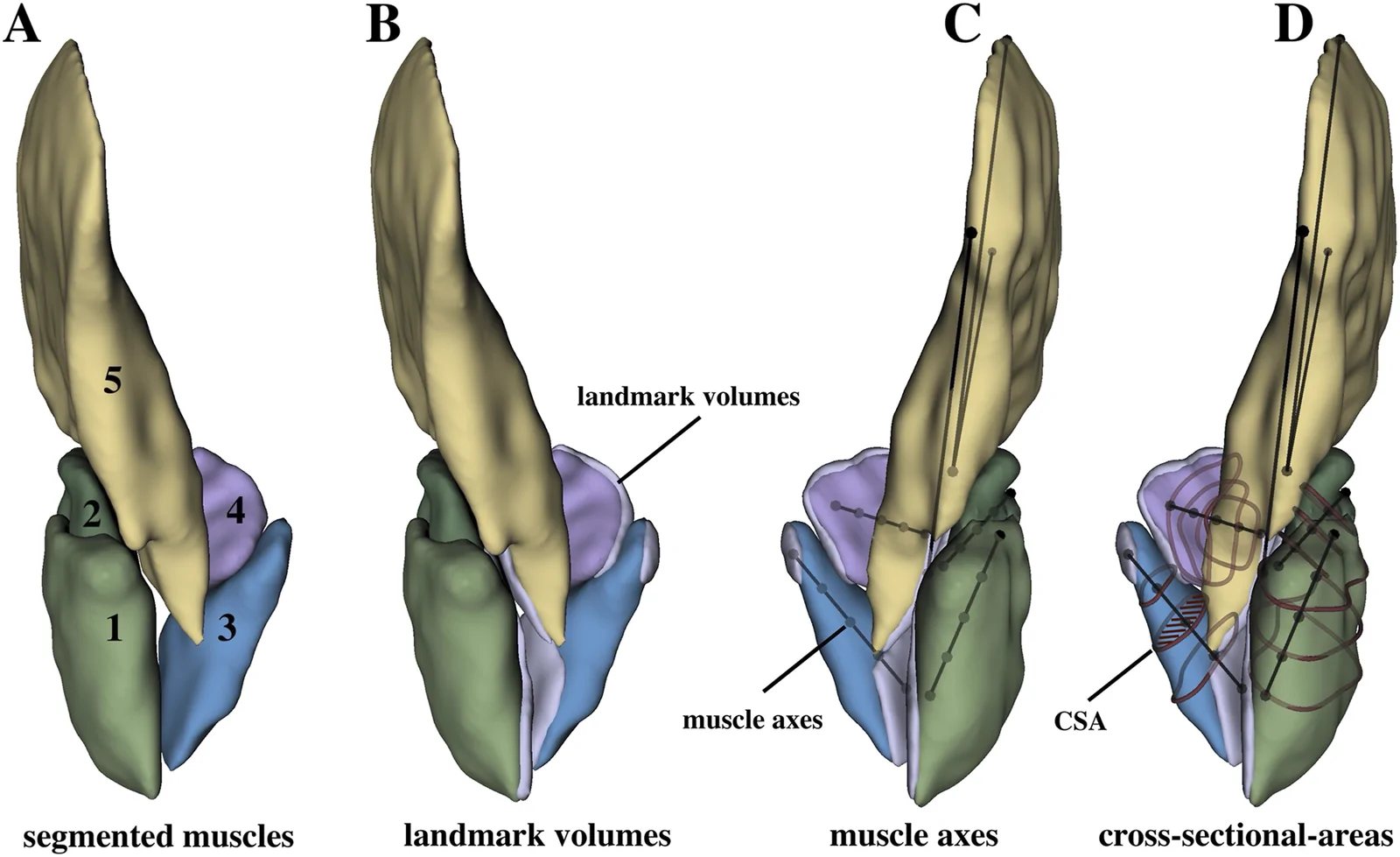
Characterization process of the masticatory muscles. **(A)** Semi-automatic segmentation of the masticatory muscles. **(B)** Center-of-mass-based definition of muscle origins and insertions. **(C)** Definition of individual muscle axes. **(D)** Standardized anatomical cross-sectional areas at 25%, 50%, and 75% of individual muscle length. Abbreviations. Cross-sectional area (CSA); M. masseter superficialis (1); M. masseter profunda (2); M. pterygoideus medialis (3); M. pterygoideus lateralis (4); M. temporalis (5).

### 2.4 Muscle measurements

After evaluating the volume and surface area values of the segmented muscles in 3D Slicer, the surface-to-volume ratio of the segments was calculated to assess the geometric compactness and asymmetries between the right and left muscles of the masticatory system across the male and female cohorts.

#### 2.4.1 Anatomical landmark definition

Muscle landmarks are defined as specific anatomical points or structures of a muscle (origins and insertions) that serve as reference points for describing its position, orientation and geometry. The proper identification and definition of landmarks for muscles are crucial for creating biomechanical models and provide information about how muscles transmit force to connected bones. Additionally, the muscle lengths and force vectors are determined by the origin and insertion landmarks. Figure 1 shows the segmented masticatory muscles, the determined muscle axes using the coordinates of the landmarks, and the standardized cross-sectional area measurements.

To accurately and consistently identify the specified landmarks, this study used a modified version of the method described by Merema et al. (2022), referred to as the center-of-mass-based segmentation method. This method identified contact areas between muscles and bones to calculate the geometric force attachment points. In this study, muscle attachment points at large muscle-bone attachment areas were defined by segmenting thin, layer-like volumes and determining their geometric centers-of-mass. Segmentation was performed by manually marking the visible central transition zone between muscle and bone in the anatomical planes. A 2.5 mm-diameter marking tool was used to trace the visible muscle-bone contact line, starting from the coronal plane, with the tool positioned centrally between the muscle and bone along the contact line. After manually segmenting the contact volumes in the coronal plane, the segmented areas were reviewed for artifacts in the axial and sagittal planes and revised as needed. The three-dimensional centroids were determined using the contact volumes and the “Centroid” function in the “Label Map Statistics” module of the imaging computing platform. This approach was employed to determine the origins and insertions of muscles that do not originate or insert at defined bone edges according to the literature (Baron and Debussy, 1979), but rather on larger bone surfaces where clear, reproducible landmarks were difficult to identify: insertion of the MMS and MMP, origin and insertion of the medial and lateral pterygoid muscles, and the insertion of the anterior part of the temporal muscle. Based on the different anatomical muscle origins and insertions, three types of landmark definitions were used in this study. Origins and insertions that could be assigned to identifiable bone structures or bony margins, according to the literature (Baron and Debussy, 1979), were defined as literature-based origins (OL) and literature-based insertions (IL). Muscles that originate from or attach to larger bone surfaces and do not exhibit distinct identifiable bone landmarks were determined using center-of-mass-based origins (OC) and center-of-mass-based insertions (IC). For the MT, the origins were defined based on the most prominent muscle points in the respective anatomical directions and designated as anterior origin (OA), medial origin (OM), and posterior origin (OP) of the MT. Figure 2 shows the anatomical locations corresponding to the landmark definitions used in this study.

**FIGURE 2:**
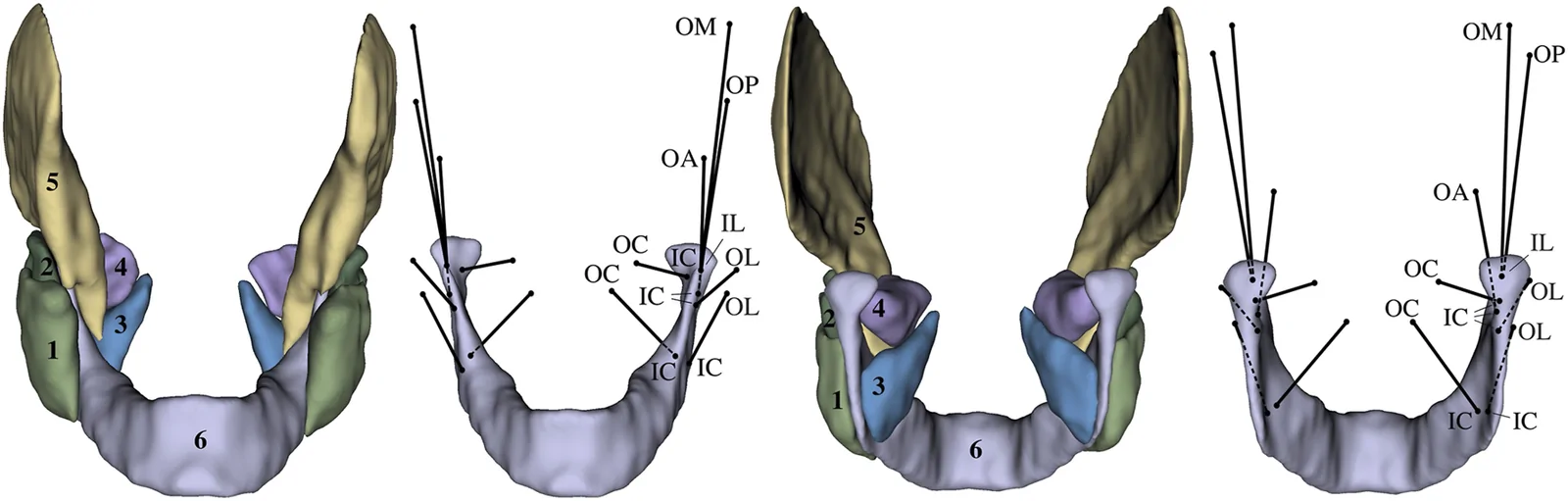
Determination of origins and insertions of the masticatory muscles. Abbreviations. M. masseter superficialis (1); M. masseter profunda (2); M. pterygoideus medialis (3); M. pterygoideus lateralis (4); M. temporalis (5); mandibula (6); origin of most prominent anterior muscle point (OA); origin of most prominent medial muscle point (OM); origin of most prominent posterior muscle point (OP); origin and insertion determined using the center-of-mass-based segmentation method (OC, IC); origin and insertion defined by bone edges according to the literature (OL, IL).

The MMS and MMP originate at the arcus zygomaticus, which can be identified in MR images based on the bony structure (OL). According to Baron and Debussy (1979), the origin of the MMS is located at the center of the anterior front two-thirds of the length of the arcus zygomaticus, while the MMP originates at the center of the posterior third of the arcus zygomaticus. This anatomical classification of the landmarks is based on established anatomical definitions and serves as the basis for reproducibility and comparability of MM measurements (Baron and Debussy, 1979). The insertion landmarks for both the MMS and MMP are attached to the lateral surface of the mandible and are measured using the center-of-mass-based segmentation method described above (IC).

According to the literature, the landmarks of the MPM and MPL are not located at specific bone edges and were therefore measured using the volume-based center-of-mass method (OC, IC). According to Baron and Debussy (1979), the medial pterygoid muscle originates from the medial part of the pterygoid fossa of the sphenoid bone and inserts on the lower, medial surface of the mandibular ramus. The origin of the lateral pterygoid muscle is located at the lateral surface of the lateral pterygoid plate, while the insertion area is on the front, anterior surface of the mandible and the joint capsule of the temporomandibular joint (Baron and Debussy, 1979). The anatomical description of the musculoskeletal geometry was also adopted in established literature, such as Peck et al. (2000), who developed a dynamic, three-dimensional mathematical model of the craniomandibular system.

For the MT, the origin is located in the fossa temporalis, which is unclear to define in the MR images because of the lack of a prominent bony edge. Additionally, the MT is a complex, fan-shaped muscle system, largely divided into subsegments according to literature-based methods for assessing MT landmarks. Baron and Debussy (1979) defined the origins of the MT using geometric measurements of four sub-segments, while other studies, such as Merema et al. (2022) only calculated a single force attachment point as the origin for the entire temporalis muscular system. Studies such as Abdi et al. (2020), Hannam et al. (2008), Peck et al. (2000), and Sagl et al. (2019) subdivided the M. temporalis into three functional parts (anterior, MTA; medial, MTM; and posterior, MTP) using imaging methods, which were also applied in this study. The origins of the MT remain undefined in the literature due to significant methodological differences. In this study, the origins of the three parts of the MT were defined by the most prominent muscle points in the anterior (OA), medial (OM), and posterior directions (OP) from the sagittal plane. According to Peck et al. (2000), the insertions of the MTM and MTP are at the apex of the processus coronoideus mandibulae, which was readily identifiable visible in the MRI datasets on both coronal and sagittal planes (IL). The insertion of the MTA is at the surface of the processus coronoideus. Therefore, the insertion point was assessed using the center-of-mass-based method described in this study and is located below the defined insertion points of the MTM and MTP (IC).

#### 2.4.2 Muscle axis and anatomical cross-sectional area measurement

In addition to evaluating the origin and insertion points of the masticatory muscles based on the literature and the center-of-mass method reported in this study, the landmarks were connected by lines to define the individual muscle axes. For a standardized definition of CSA, three additional measurement points at equal distances along the muscle axis from origin to insertion were applied: the first measurement point at 25%, the second at 50%, and the third at 75% of the muscle axis length (Figure 1). These predefined, standardized CSA levels were used as morphometric parameters to assess comparisons by sex and regional variation in muscle shape along the functional muscle axis. Additional intermediate measurements and maximum CSA approach were therefore not applied, as the aim was to use three standardized measurement levels to descriptively assess muscle shape and to compare reproducible CSA levels across the cohorts.

To obtain accurate values for the anatomical CSAs, the measurement planes of the MMS and MMP, as well as the MPM and MPL, had to be reformatted orthogonally to the muscle axes in the 3D Slicer imaging platform. After reformatting, each plane was aligned at the previously defined 25%, 50%, and 75% points of the muscle lengths. Then, the cross-sectional areas were measured using the “Closed Curve” markup module in 3D Slicer. This process was applied to all segmented masticatory muscles of men and women, except for the MT (Figure 1). It allows assessments of morphological changes along the muscle axes from origin to insertion and provides information on sex differences and asymmetries between the right and left muscles within the cohorts. No measurement points and CSAs were calculated for the MT due to its complex muscular system, its division into anterior, medial, and posterior parts, and the varying pennation angles of its muscle fibers. Since the CSA measurement planes in this study were defined orthogonally to the individual axes, the complex subdivision of the MT limited the reproducible definition of standardized CSA levels; therefore, CSA measurements were not applied to the MT.

### 2.5 Statistical analysis and comparison

The absolute measured values of all muscle parameters (length, CSA, volume, surface, surface-to-volume ratio) were recorded separately for men (sample size (*n*) = 7) and women (*n* = 15). Furthermore, the normality of the distributions was assessed using the Shapiro-Wilk test: both participant groups (female and male) showed significant deviations from normality (*p* < 0.05), so only non-parametric tests were conducted in RStudio (version 2026.01.0 + 392, Posit PBC, Boston, MA. URL www.posit.co). This approach was also considered appropriate because of the unequal sample sizes between male and female participants. For each combination of muscle, affected muscle side, and parameter, the sample size (*n*), minimum (Min), maximum (Max), first quartile (*Q1*), median (*Q2*), third quartile (*Q3*), and IQR were calculated. Supplementary Tables S1, S2 show the side-specific statistical analysis of the masticatory muscles in men and women, whereas Supplementary Tables S3, S4 show the combined (bilateral) statistical analysis of the masticatory muscles in men and women.

To examine deviations in muscle parameters between female and male datasets, the Wilcoxon Rank-Sum test for independent samples was applied, as this non-parametric test is suitable for comparing two independent groups with unequal sample sizes. Due to the large number of tests conducted for all muscle parameters, the False-Discovery-Rate (FDR) correction was applied using the Benjamini-Hochberg method. FDR corrected *p*-values (*p*_BH_) at or below 0.05 were considered statistically significant. Additionally, the Rank-Biserial correlation (effect size for the Wilcoxon Rank-Sum test) was calculated to quantify the magnitude and direction of sex-related cohort differences: positive effect sizes up to +1 indicate higher measurements in men, while negative effect sizes down to −1 indicate higher measurements in women. Effect sizes were interpreted according to the following thresholds: small *r* ≥ 0.1, medium *r* ≥ 0.3, large *r* ≥ 0.5.

To account for the small and unequal sample sizes, the sex-specific Wilcoxon Rank-Sum analysis was supplemented by a simulation-based *post hoc* power analysis. The observed male and female datasets were resampled with replacement using the original group sizes (men: *n* = 7; women: *n* = 15). In each of 10,000 simulations, the same Wilcoxon Rank-Sum testing procedure was repeated for all sex-specific muscle parameters, followed by Benjamini-Hochberg correction of the resulting *p*-values. Statistical power was defined as the proportion of simulations in which the FDR-adjusted *p*-value remained significant at *p*_BH_ ≤ 0.05. Power values ≥0.80 were considered sufficient, whereas values below 0.80 were interpreted as indicating limited statistical power.

Differences between the right and left muscles were analysed separately for women and men using the Wilcoxon Signed-Rank test on 34 paired parameters. Significant results were reviewed using the FDR correction at a significance level of *p*_BH_ ≤ 0.05. To quantify potential muscle side effects, the effect sizes (*r*) were calculated for all paired tests. The following effect size thresholds were used to describe the magnitude of muscle side effects of the Wilcoxon Signed-Rank test: small *r* ≥ 0.1, medium *r* ≥ 0.3, large *r* ≥ 0.5.

To assess intra-observer reliability, four MRI datasets, comprising two female and two male participants, were independently re-segmented and re-measured by the same investigator using the identical segmentation, landmark definition, and measurement procedures. The repeated analysis was performed after an interval of 5–7 months. Intra-observer reliability was calculated separately for all 68 morphometric parameters using an intraclass correlation coefficient based on a two-way mixed-effects model for absolute agreement and single measurements, ICC(A,1), with 95% confidence intervals. ICC values were interpreted as poor (<0.50), moderate (0.50–0.74), good (0.75–0,89), and excellent (>0.90). Because the repeated-measurements analysis included only four datasets, the ICC estimates were interpreted cautiously.

To contextualize the muscle parameters determined in this study using the semi-automatic, center-of-mass-based segmentation method, the measured muscle volumes and lengths were compared with values from established literature, which employ cadaveric (Koolstra and Van Eijden, 1997) and imaging-based methodologies (Adisen et al., 2018; Ng H. et al., 2009; Lin et al., 2025; Heins et al., 2025). No formal statistical comparisons were performed between the present dataset and previously published data; therefore, these comparisons were interpreted as descriptive observations. Volume and length were selected as the primary comparison parameters because they form the basis for calculating the measurement points along the muscle axis and the anatomical cross-sectional areas.

## 3 Results

### 3.1 Segmentation and quantification process

The semi-automatic method (Grow from Seeds) in 3D Slicer was applicable for all muscle groups examined (MMS, MMP, MPM, MPL, and MT). The muscle boundaries of the MM and MT were more readily identifiable due to their proximity to prominent bony structures, whereas the segmentation of the medial and lateral pterygoid muscles proved challenging. Smaller contrast differences at the muscle margins complicated the semi-automatic segmentation, necessitating more intensive manual post-processing of the muscle segments.

When manually defining the landmarks of the masticatory muscles using the center-of-mass-based segmentation method, differences in identifiability were observed depending on the anatomical location. The insertions of all the muscles examined on the mandible (MMS, MMP, MPM, MPL, and MT) were more consistently delineated because of the bony structure on MR images. In comparison, the origins of the pterygoid muscle parts showed only minimal contrast differences to the related bone surface, making the contact volume of the muscle origins more difficult to segment visually and resulting in greater time expenditure. Landmarks, which are located at prominent bony margins according to the literature were more readily identifiable on the MR slices and could be reproducibly marked.

Plotting the respective functional muscle axes between the calculated centers-of-mass of the origin and insertion volumes proceeded without major difficulties. However, determining the cross-sectional areas required additional methodological effort because they had to be manually marked along the individual muscle boundaries.

### 3.2 Muscle geometry measurements

Analysis of the muscle geometry shows that the lengths of the masticatory muscles differ noticeably. The MMS has the greatest longitudinal extent, while the MMP is slightly shorter. In terms of length, the MT is by far the longest of all masticatory muscles, with the posterior fibers representing the longest individual stretch compared to the medial and anterior fibers. The medial and lateral pterygoid muscles lie, in terms of total length, between the two parts of the MM, with the MPL being the shorter of the 2 M. pterygoideus (MP) units.

Regarding cross-sectional area trends, the MMS CSA increases continuously from its origin to its insertion and reaches its maximum among the three predefined points at 75% of the muscle’s length, where it widens toward its insertion point on the mandibular ramus. In the MMP, the largest CSA values occur between points 1 and 2 (25% and 50% of the distance between origin and insertion), followed by a decrease in cross-sectional area toward the insertion. The medial pterygoid muscle also shows a steady increase in CSA along its functional axis from 25% to 75%, whereas the lateral pterygoid muscle’s CSA remains virtually constant over its entire length.

An analysis of the volume data reveals that the MT is the largest muscle in the masticatory system in both male and female participants and has the greatest surface area. The total volume of the MM is determined almost exclusively by the superficial portion, which exceeds the volume of the MMP. The medial and lateral pterygoid muscles contribute substantially to the total volume of the masticatory musculature, with the lateral pterygoid muscle showing only minor differences in volume compared to the medial pterygoid muscle.

The surface-to-volume ratios reflect the different muscular characteristics. The MMS and the MPL exhibit low ratios compared to their corresponding muscle components (MMP and MPM), indicating a more compact, thicker muscle morphology. In contrast, the MMP and the MPM muscle show higher surface-to-volume ratios, which characterize thinner, flatter muscle structures.

### 3.3 Morphological characterization

The Wilcoxon Rank-Sum test indicates that 66 of 68 independent muscle parameters differ significantly between female and male participants, even after FDR correction (*p*_BH_ ≤ 0.05). Only the CSA at 25% of the distance between the origin and insertion of the right and left MMS show no significant differences between the male and female datasets (Table 1). The Rank-Biserial effect size emphasizes morphological sex differences: 58 out of 68 parameters show positive effect sizes and therefore higher values in men; 57 of these 58 muscle parameters demonstrate large effects (*r* ≥ 0.5), with one indicating a medium effect (*r* ≥ 0.3). Conversely, 10 of 68 parameters have higher values in women. These were exclusively related to the surface-to-volume ratio and demonstrate high effect sizes (Table 1). The simulation-based *post hoc* power analysis showed power values ranging from 0.324 to 1.000. Overall, 45 of 68 comparisons reaches sufficient statistical power (Power ≥0.80), while 23 comparisons showed limited power.

**TABLE 1:** Wilcoxon Rank-Sum test for sex differences in masticatory muscle parameters with FDR corrected *p*-values (*p*_BH_), Rank-Biserial effect sizes (*r*), and simulation-based *post hoc* power values.

​	​	Right	​	​	Left	​	​
Muscle	Parameter	*p*_BH_	*r*	Power	*p*_BH_	*r*	Power
MMS	Length	0.004	0.87	0.977	0.005	0.85	0.950
​	CSA 25%	**0.130** ^**a**^	0.42	0.324	**0.068** ^**a**^	0.50	0.454
​	CSA 50%	0.017	0.68	0.729	0.037	0.58	0.585
​	CSA 75%	0.005	0.86	0.978	0.015	0.70	0.789
​	Volume	0.003	0.92	0.999	0.004	0.87	0.988
​	Surface	0.004	0.90	0.996	0.005	0.85	0.979
​	Surface/Volume ratio	0.018	**−0.67** ^**b**^	0.747	0.013	**−0.72** ^**b**^	0.861
MMP	Length	0.012	0.73	0.861	0.010	0.75	0.900
​	CSA 25%	0.017	0.68	0.777	0.028	0.62	0.649
​	CSA 50%	0.007	0.79	0.923	0.015	0.70	0.770
​	CSA 75%	0.042	0.56	0.550	0.033	0.60	0.636
​	Volume	0.003	0.94	0.999	0.004	0.87	0.983
​	Surface	0.003	0.96	1.000	0.006	0.81	0.932
​	Surface/Volume ratio	0.023	**−0.64** ^**b**^	0.675	0.013	**−0.71** ^**b**^	0.750
MPM	Length	0.005	0.83	0.967	0.013	0.71	0.807
​	CSA 25%	0.009	0.76	0.894	0.005	0.84	0.978
​	CSA 50%	0.004	0.87	0.986	0.006	0.81	0.947
​	CSA 75%	0.005	0.84	0.970	0.005	0.83	0.942
​	Volume	0.004	0.90	0.997	0.005	0.85	0.980
​	Surface	0.004	0.90	0.998	0.006	0.81	0.954
​	Surface/Volume ratio	0.005	**−0.85** ^**b**^	0.979	0.017	**−0.68** ^**b**^	0.755
MPL	Length	0.037	0.58	0.587	0.042	0.56	0.540
​	CSA 25%	0.005	0.86	0.978	0.003	0.98	1.000
​	CSA 50%	0.003	1.00	1.000	0.003	0.98	1.000
​	CSA 75%	0.003	1.00	1.000	0.003	1.00	1.000
​	Volume	0.003	0.96	1.000	0.003	0.98	1.000
​	Surface	0.003	0.94	1.000	0.003	0.98	1.000
​	Surface/Volume ratio	0.003	**−0.96** ^**b**^	1.000	0.003	**−0.94** ^**b**^	1.000
MTA	Length	0.023	0.64	0.699	0.037	0.58	0.592
MTM	Length	0.017	0.68	0.764	0.020	0.66	0.736
MTP	Length	0.008	0.77	0.900	0.006	0.81	0.946
MT	Volume	0.004	0.89	0.992	0.004	0.87	0.969
​	Surface	0.017	0.68	0.755	0.007	0.79	0.937
​	Surface/Volume ratio	0.013	**−0.71** ^**b**^	0.830	0.037	**−0.58** ^**b**^	0.580

Abbreviations. FDR corrected *p*-values (*p*_BH_); Rank-Biserial correlation (*r*); cross-sectional area (CSA); *p*_BH_ > 0.05 (a); greater values in women (b); Power, simulation-based *post hoc* statistical power; M. masseter superficialis (MMS); M. masseter profunda (MMP); M. pterygoideus medialis (MPM); M. pterygoideus lateralis (MPL); M. temporalis anterior (MTA), M. temporalis medial (MTM); M. temporalis posterior (MTP).

In men, the Wilcoxon Signed-Rank test shows no significant difference across all 34 paired parameters at a significance level of *p* ≥ 0.05. The findings are confirmed using the FDR correction (*p*_BH_ ≥ 0.05). Rank-Biserial effect sizes indicate 14 small effects (*r* ≥ 0.1), eight medium effects (*r* ≥ 0.3), and 12 large effects (*r* ≥ 0.5) between the right and left muscle sides in men. Female results of the Wilcoxon Signed-Rank test show no significant difference between the right and left muscles in all 34 paired parameters with a significance level of *p* ≥ 0.05. The FDR correction supports the results of the non-parametric test and confirms no significant differences between the muscle sides in female participants. Effect sizes from the Rank-Biserial correlation suggest mostly minor asymmetries: 27 out of 34 parameters demonstrate small effects (*r* ≥ 0.1), seven show medium effects (*r* ≥ 0.3), and none indicate large deviations (*r* ≥ 0.5) between the right and left muscles in women. The comparison of the masticatory muscle sides is shown in Table 2.

**TABLE 2:** Wilcoxon Signed-Rank test for comparison between left and right muscles of the masticatory muscles with FDR corrected *p*-values (*p*_BH_) and Rank-Biserial effect sizes (*r*).

​	​	Male	​	Female	​
Muscle	Parameter	*p*_BH_	*r*	*p*_BH_	*r*
MMS	Length	0.31	**0.77** ^a^	0.76	**0.30** ^b^
​	CSA 25%	0.89	0.06	0.81	0.11
​	CSA 50%	0.31	**0.70** ^a^	0.83	0.10
​	CSA 75%	0.50	**0.45** ^b^	0.81	0.14
​	Volume	0.31	**0.77** ^a^	0.91	0.04
​	Surface	0.50	**0.45** ^b^	0.83	0.08
​	Surface/Volume ratio	0.83	0.20	0.81	0.12
MMP	Length	1.00	0.00	0.76	0.25
​	CSA 25%	0.74	0.26	0.81	0.13
​	CSA 50%	0.89	0.06	0.77	0.18
​	CSA 75%	0.74	0.26	0.76	**0.39** ^b^
​	Volume	0.68	**0.32** ^b^	0.76	0.27
​	Surface	0.50	**0.51** ^a^	0.76	0.26
​	Surface/Volume ratio	0.83	0.19	0.76	**0.33** ^b^
MPM	Length	0.31	**0.70** ^a^	0.76	**0.34** ^b^
​	CSA 25%	0.50	**0.45** ^b^	0.76	0.24
​	CSA 50%	0.88	0.16	0.76	**0.34** ^b^
​	CSA 75%	0.31	**0.77** ^a^	0.83	0.08
​	Volume	0.31	**0.70** ^a^	0.76	0.27
​	Surface	0.50	**0.51** ^a^	0.77	0.18
​	Surface/Volume ratio	0.50	**0.51** ^a^	0.76	**0.43** ^b^
MPL	Length	0.48	**0.57** ^a^	0.77	0.18
​	CSA 25%	0.62	**0.38** ^b^	0.85	0.07
​	CSA 50%	0.89	0.06	0.81	0.12
​	CSA 75%	0.31	**0.90** ^a^	0.76	0.23
​	Volume	0.89	0.12	0.76	0.24
​	Surface	0.89	0.13	0.76	0.26
​	Surface/Volume ratio	0.64	**0.36** ^b^	0.77	0.18
MTA	Length	0.74	0.26	0.76	0.24
MTM	Length	0.89	0.06	0.76	0.23
MTP	Length	0.68	**0.32** ^b^	0.92	0.03
MT	Volume	0.48	**0.57** ^a^	0.77	0.18
​	Surface	0.89	0.06	0.81	0.11
​	Surface/Volume ratio	0.50	**0.45** ^b^	0.76	**0.46** ^b^

Abbreviations. cross-sectional area (CSA); FDR corrected *p*-values (*p*_BH_); large effects (*r* ≥ 0.5) (a); medium effects (*r* ≥ 0.3) (b); M. masseter superficialis (MMS); M. masseter profunda (MMP); M. pterygoideus medialis (MPM); M. pterygoideus lateralis (MPL); M. temporalis anterior (MTA), M. temporalis medial (MTM); M. temporalis posterior (MTP).

Intra-observer reliability was assessed for all 68 morphometric parameters in four independently resegmented and re-measured datasets. The overall median ICC was 0.866, with parameter-specific values ranging from 0.126 to 0.9957. The median ICC was 0.817 for muscle length measurements, 0.848 for cross-sectional area measurements, 0.949 for muscle volume measurements, 0.919 for surface area measurements, and 0.899 for surface-to-volume ratios. The corresponding parameter-specific ICC ranges are presented in Table 3. Overall, the results indicated predominantly good to excellent intra-observer reliability.

**TABLE 3:** Intra-observer reliability of morphometric parameter groups of the masticatory muscles.

Parameter group	Number of parameters	Median ICC	MIN	MAX
Length	14	0.817	0.126	0.986
CSA	24	0.848	0.560	0.973
Volume	10	0.949	0.803	0.974
Surface	10	0.919	0.655	0.968
Surface-to-volume ratio	10	0.899	0.795	0.995
Overall	68	0.866	0.126	0.995

Abbreviations. Cross-sectional area (CSA); intraclass correlation coefficient for absolute agreement based on single measurements (ICC); minimum (MIN) and maximum (MAX) parameter-specific ICC values.

### 3.4 Comparison with previous studies

The total volumes of male and female participants determined in this study are consistently higher than those reported in the cadaver literature (Van Eijden et al., 1997). The mean values for contractile muscle volume reported in the literature represent the lowest values among the reference studies consulted in a direct volume comparison and are mostly within the range or below the female medians. The volume data reported by Heins et al. (2025) is also in the lower range of the literature values, particularly for the MMS and MMP, and remain below the male and female medians reported in this study. In contrast, the mean values with standard deviation for the MM, as well as the MMS and MMP, and the median with interquartile range for the pterygoid muscle reported by Lin et al. (2025) largely align with the interquartile ranges of the male volumes recorded in this study, although the dispersion and the interquartile range of the volume values reported in the Literature are higher than the interquartile range of the data according to this study.

The mean values of the measured volumes reported by Ng H. et al. (2009) for the MM and the medial and lateral pterygoid muscles are higher than the mean values reported by Van Eijden et al. (1997) and lower than the mean and median values reported by Lin et al. (2025), although the values reported by Ng H. et al. (2009) also exceed the literature values reported by Van Eijden et al. (1997) for the volume of the MT. Furthermore, the mean values reported by Ng H. et al. (2009) fall between the medians for men and women reported in this study, with the mean values from the literature for the volume of the medial pterygoid muscle and the MT showing greater agreement with the female data from this study, and the mean values for the volume of the MM and the lateral pterygoid muscle from the literature lying between data for women and men from this cohort. The descriptive mean data on total muscle volumes in women, especially for the MM and MP muscles, reported by Adisen et al. (2018) are above the median values for women in this study and show a substantially higher variation in values around the mean compared to the interquartile ranges from this cohort.

In contrast to the volume data, the descriptive data for muscle lengths according to Van Eijden et al. (1997) tend to fall within the range of the male data collected in this study, although the variation in length data around the means is considerably higher than the interquartile ranges of the female and male cohorts in this study.

#### 3.4.1 Volume of M. masseter

Mean values of the MM according to Ng H. et al. (2009) for the right (29.65 ± 6.77 cm^3^) and left (29.54 ± 5.19 cm^3^) muscles fall within the range between the medians of males (right: 34.99 cm^3^; left: 33.94 cm^3^) and females (right: 24.44 cm^3^; left: 23.63 cm^3^) volumes from this study and indicate agreement between the data sets. The mean value reported by Lin et al. (2025) for MM volume (36.40 ± 8.06 cm^3^) is slightly above the medians for both male muscle sides, making it substantially higher than the corresponding female median values and the mean values for the right and left muscles according to Ng H. et al. (2009). For the total volume of the MM in women, the mean value reported by Adisen et al. (2018), 54.90 ± 13.40 cm^3^, differs from the median collected in this study (47.49 cm^3^; *Q1* = 45.67 cm^3^, *Q3* = 51.67 cm^3^) (Figure 3; Supplementary Table S5).

**FIGURE 3:**
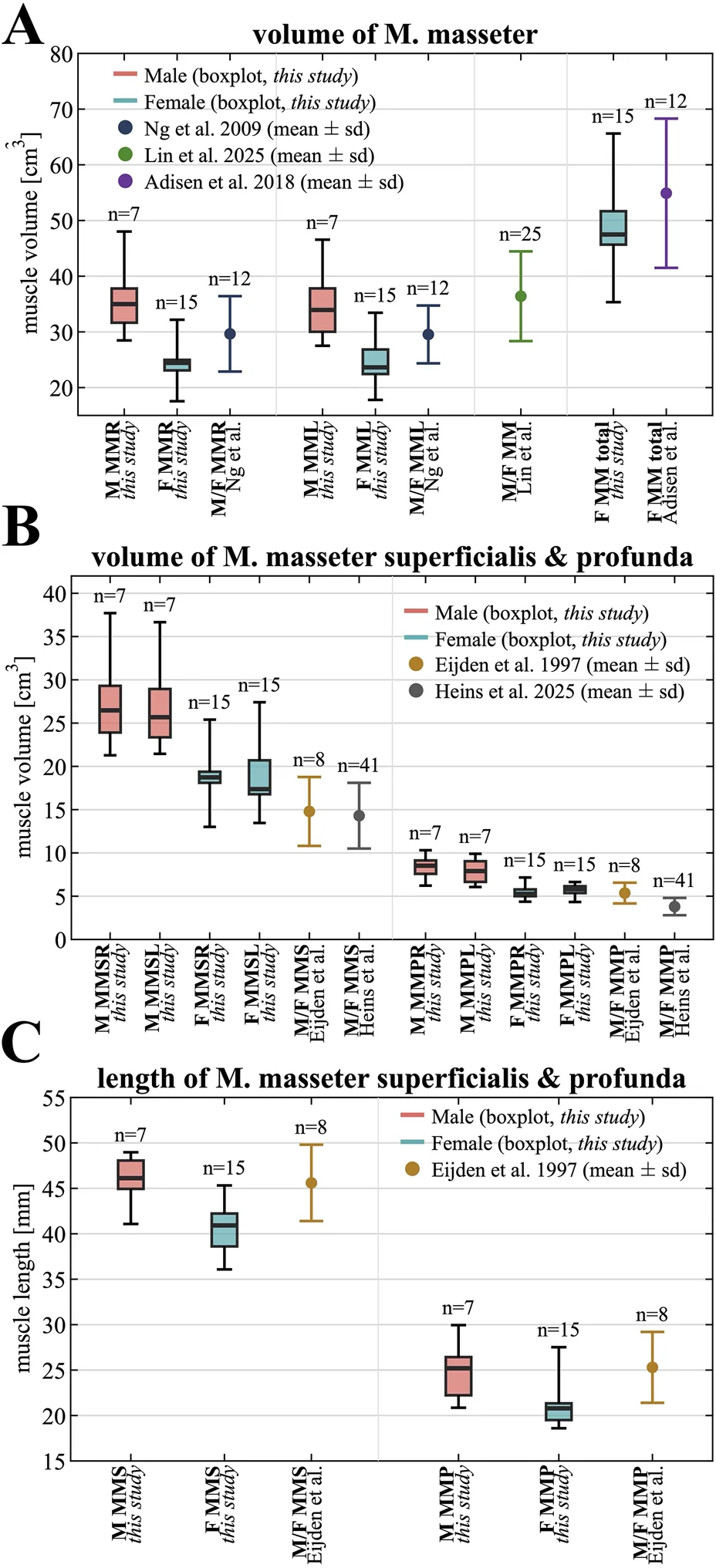
M. masseter volume and length comparison. **(A)** M. masseter volume comparison. **(B)** M. masseter superficialis and M. masseter profunda volume comparison. **(C)** M. masseter superficialis and M. masseter profunda length comparison. Abbreviations. Male (M); female (F); male/female (M/F); M. masseter right (MMR); M. masseter left (MML); M. masseter (MM); M. masseter superficialis right (MMSR); M. masseter superficialis left (MMSL); M. masseter superficialis (MMS); M. masseter profunda right (MMPR); M. masseter profunda left (MMPL); M. masseter profunda (MMP).

For the superficial muscle, the median values obtained for males (26.47 cm^3^ on the right, 25.68 cm^3^ on the left) are noticeably higher than the contractile muscle volume of 14.79 cm^3^ ± 3.98 cm^3^ reported by Van Eijden et al. (1997). The mean value reported by Heins et al. (2025) for the MMS is nearly identical to the data reported by Van Eijden et al. (1997), with a mean volume of 14.80 cm^3^ and a standard deviation of 3.80 cm^3^, and is therefore below the male and female medians of this study. The medians of the female data collected in this study, with 18.73 cm^3^ (IQR: 18.10–19.38 cm^3^) on the right and 17.37 cm^3^ (IQR: 16.78–20.70 cm^3^) on the left, are slightly above the volume of the MMS muscle reported by Van Eijden et al. (1997) in their cadaver study. Although the upward dispersion of the mean value reported in the literature is slightly above the medians, it remains within the interquartile range of the descriptive statistics from the female data sets for both muscle sides in this study (Figure 3; Supplementary Table S5).

The male medians for the MMP (right 8.52 cm^3^, left 7.90 cm^3^) are substantially higher than the mean value (5.36 ± 1.20 cm^3^) according to the literature, while the female medians (right 5.27 cm^3^, left 5.81 cm^3^) are consistent with the mean value (Van Eijden et al., 1997). The mean value reported by Heins et al. (2025) for the MMP, with a mean volume of 3.80 ± 1.00 cm^3^, is lower than the reported mean value by Van Eijden et al. (1997) and therefore substantially lower than the male and female medians determined in this study. The upper standard deviation of the contractile muscle volume reported by Van Eijden et al. (1997), with a value of 5.36 ± 1.20 cm^3^, lies above the interquartile range of female data for right (5.00–5.78 cm^3^) and left side of the MMP (5.36–6.15 cm^3^) and is slightly below the first quartile of the male data (right 7.58 cm^3^, left 6.65 cm^3^) with respect to this study (Figure 3; Supplementary Table S5).

#### 3.4.2 Length of the M. masseter

The length of the MMS, with a mean value reported by Van Eijden et al. (1997) at 45.60 ± 4.20 mm, is slightly below the median of 46.12 mm recorded in this study and above the median for women, which is 40.92 mm. The lower standard deviation of the MMS length in the mean value lies below the third quartile of the values for women and is thus within the interquartile range of the female dataset (Van Eijden et al., 1997). The mean length of the deep masseter muscle, according to Van Eijden et al. (1997) at 25.30 ± 3.90 mm, is nearly identical to the male median of 25.20 mm (IQR: 22.23–26.43 mm) and is above the female median in this study, which is 20.79 mm (IQR: 19.50–21.34 mm). The lower standard deviation of the reported mean length, 25.30 ± 3.90 mm, is at the third quartile of the female dataset for the deep masseter muscle in this study (Van Eijden et al., 1997). The comparison of the MM volume and length with previous studies is shown in Figure 3 and Supplementary Table S5.

#### 3.4.3 Volume of M. pterygoideus

The mean values of the MPM according to Ng H. et al. (2009) (right 8.92 ± 1.54 cm^3^, left 8.69 ± 1.66 cm^3^) are slightly above the calculated female medians (right 7.66 cm^3^, left 8.52 cm^3^) for the MPM muscle and are considerably below the male medians (right 13.76 cm^3^, left 12.64 cm^3^). The mean contractile muscle volume by Van Eijden et al. (1997) is 7.73 ± 2.12 cm^3^, which falls between the female median values for the volume of the MPM and below the male volume values. Heins et al. (2025) reported a slightly higher mean value for the MPM than Van Eijden et al. (1997), with a mean volume of 8.20 cm^3^, and is therefore approximately at the level of the female medians reported in this study, while remaining below the male medians. The median reported by Lin et al. (2025), at 12.19 cm^3^, lies slightly below the medians of the male data in this study and shows a larger interquartile range of 11.09–13.93 cm^3^, which corresponds to the male data’s interquartile range (*Q1*-*Q3*). The total volume of the MPM in women in this study shows a median of 16.33 cm^3^ (IQR: 15.13–18.43 cm^3^) and is slightly below the mean value reported by Adisen et al. (2018) of 18.50 ± 6.70 cm^3^, although the standard deviation of the mean reported in this literature is twice as large as the interquartile range of the data collected from the female cohort in this study (Figure 4; Supplementary Table S6).

**FIGURE 4:**
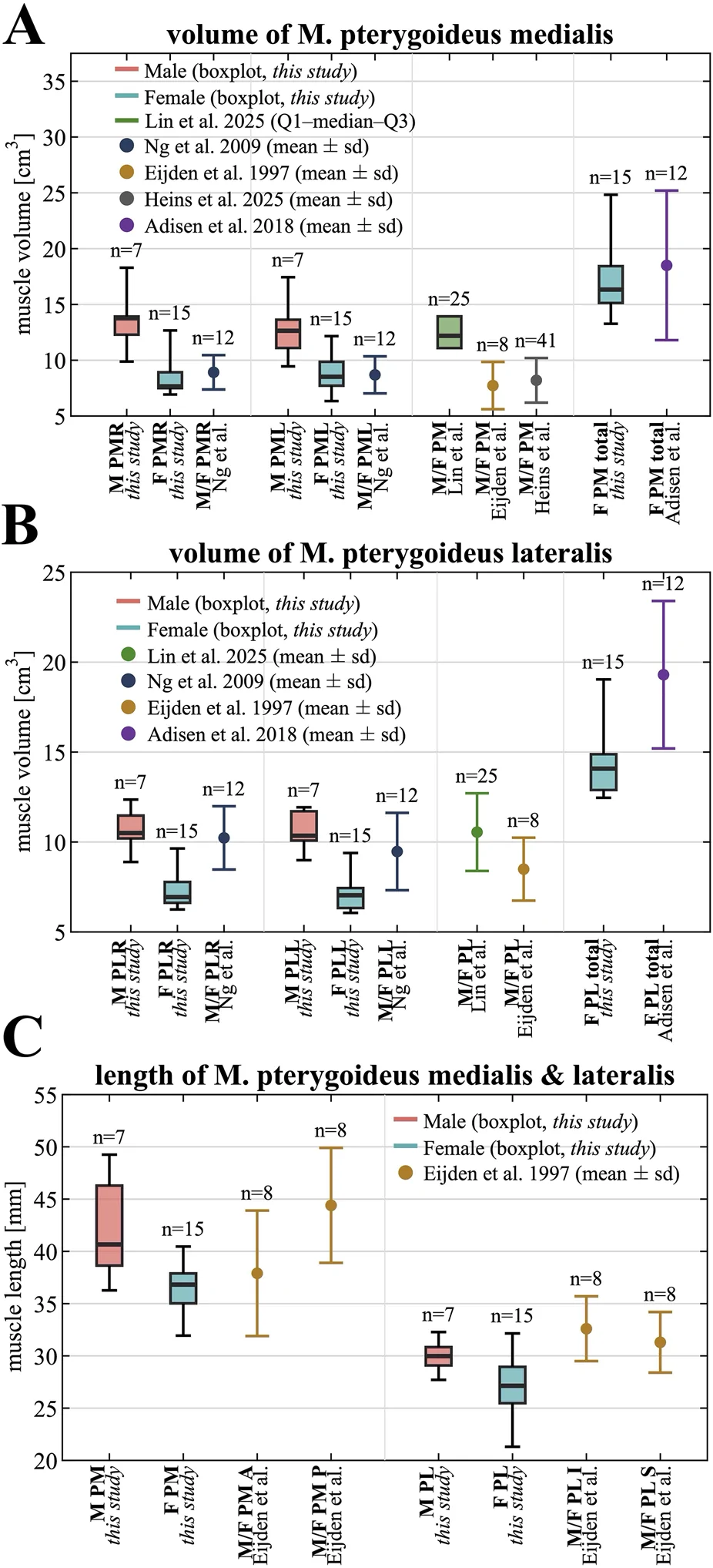
M. pterygoideus medialis and lateralis volume and length comparison. **(A)** M. pterygoideus medialis volume comparison. **(B)** M. pterygoideus lateralis volume comparison. **(C)** M. pterygoideus medialis and M. pterygoideus lateralis length comparison. Abbreviations. Male (M); female (F); male/female (M/F); M. pterygoideus medialis right (PMR); M. pterygoideus medialis left (PML); M. pterygoideus medialis (PM); M. pterygoideus lateralis right (PLR); M. pterygoideus lateralis left (PLL); M. pterygoideus lateralis (PL); M. pterygoideus medialis anterior (PM A); M. pterygoideus medialis posterior (PM P); M. pterygoideus lateralis inferior (PL I); M. pterygoideus lateralis superior (PL S).

Figure 4 shows that the mean values of the MPL from Ng H. et al. (2009) are 10.23 ± 1.76 cm^3^ on the right and 9.47 ± 2.15 cm^3^ on the left, slightly below the male medians (right 10.50 cm^3^, left 10.35 cm^3^). Furthermore, the mean values reported in the literature exceed the female medians reported in this study, with 6.94 cm^3^ on the right and 7.04 cm^3^ on the left. The mean value reported by Lin et al. (2025), at 10.55 ± 2.16 cm^3^, is exactly at the male median and thus considerably above the female medians in this study. The contractile muscle volume reported by Van Eijden et al. (1997), with a mean of 8.49 cm^3^ and a standard deviation of 1.75 cm^3^, lies below the male median and above the female median of the cohort, with the low standard deviation at the level of the female median and the upper standard deviation at the level of the male median. Regarding the total volume of the MPL, the mean value reported in the literature (19.30 ± 4.10 cm^3^) is substantially higher than the female median of 14.08 cm^3^ (IQR: 12.89–14.88 cm^3^) and exhibits a standard deviation twice as high as the interquartile range of the data from this study (Adisen et al., 2018) (Supplementary Table S6).

#### 3.4.4 Length of the M. pterygoideus

The length of the anterior part of the MPM, as reported in the literature at 37.90 ± 6.00 mm, falls between the male median of 40.66 mm (IQR: 38.64–46.29 mm) and female median of 36.82 mm (IQR: 35.03–37.89 mm), with the lower standard deviation of the mean length lying below the first quartile for women, while the upper standard deviation remains within the interquartile range for males in this study (Van Eijden et al., 1997). The mean length of the posterior part of the MPM at 44.40 ± 5.50 mm is above the male median value (Van Eijden et al., 1997), but falls within the male interquartile range (IQR: 38.64–46.29 mm) and is therefore markedly above the female median value for the length of the MPM in this study. The mean length values reported in the literature according to Van Eijden et al. (1997) for the inferior (32.60 ± 3.10 mm) and superior parts (31.30 ± 2.90 mm) are above the medians for both sexes (male 29.98 mm, female 27.14 mm). The mean length of the inferior part of the MPL is at the third quartile (30.84 mm) of the interquartile range of the male data from this study, while the lower standard deviation of the inferior part of the MPL is slightly above the third quartile of the female cohort (Van Eijden et al., 1997). The mean length of the superior part of the MPL is at the upper limit of the male interquartile range, while the lower standard deviation of the superior mean reported by Van Eijden et al. (1997) is slightly below the third quartile (28.95 mm) of the female data (Figure 4) (Supplementary Table S6).

#### 3.4.5 Volume of M. temporalis

The mean volume values from Ng H. et al. (2009) of the MT (right 48.80 ± 8.48 cm^3^, left 47.70 ± 7.37 cm^3^) are slightly above the median values for women (right 46.80 cm^3^, left 47.03 cm^3^) and noticeably below the median values for men (right 62.80 cm^3^, left 63.50 cm^3^). Although the standard deviation of the volume measurements reported in this literature for both the right and left muscles does not overlap with the interquartile ranges of the male data from this cohort. The interquartile range of women overlaps with the volume values around the mean of Ng H. et al. (2009). The mean contractile muscle volume reported by Van Eijden et al. (1997) is 34.79 ± 8.27 cm^3^, which is substantially below the medians for both male and female cohorts and below the first quartile of female volumes for both sides (41.85 cm^3^ and 43.89 cm^3^), with the upper standard deviation around the mean volume according to the literature being equivalent to the first quartiles (right 43.89 cm^3^, left 41.85 cm^3^) of the female data from this study. The literature value for the total mean muscle volume of women according to Adisen et al. (2018), at 92.40 ± 12.10 cm^3^, aligns with the data collected from the female cohort in this study, with a median of 93.83 cm^3^ (IQR: 86.23–98.42 cm^3^). Figure 5 and Supplementary Table S7 show a comparison between MT volumes reported in this study with those from the literature.

**FIGURE 5:**
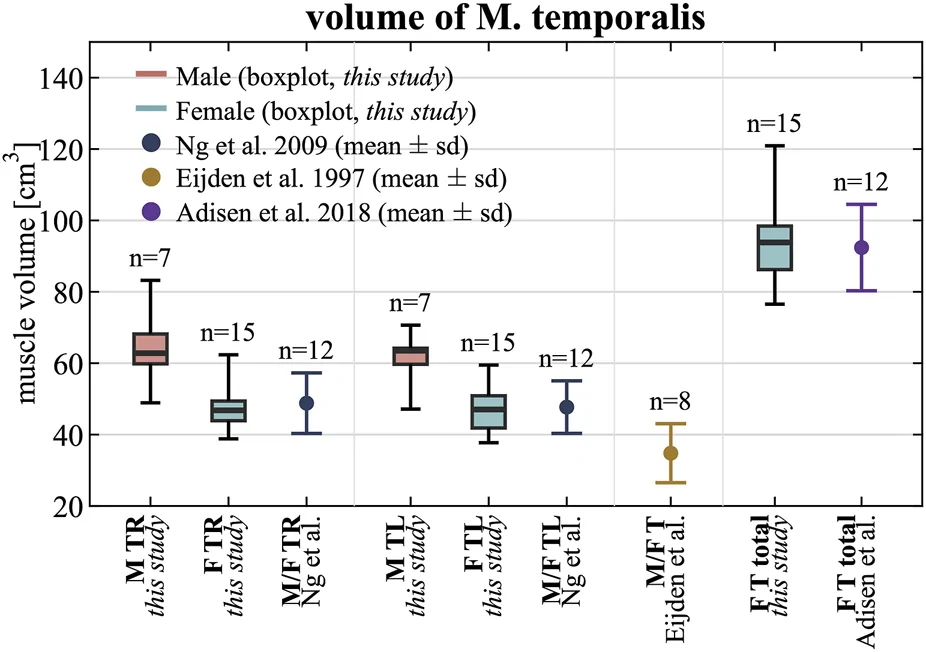
M. temporalis volume comparison. Abbreviations. Male (M); female (F); male/female (M/F); M. temporalis right (TR); M. temporalis left (TL); M. temporalis (T).

## 4 Discussion

In this study, the semi-automatic segmentation approach was applicable to all examined masticatory muscles, although the pterygoid muscles required more manual refinement due to less readily identifiable anatomical boundaries. The muscle geometry analysis revealed distinct differences in length, volume, CSA, and surface-to-volume ratio between the examined muscles, with the MT showing the largest muscle volume and surface area. Furthermore, 66 out of 68 muscle parameters indicate significant sex differences (*p*_BH_ ≤ 0.05) according to the FDR correction, with higher values in men for volumes, lengths, and the majority of cross-sectional areas. In contrast, the values in women demonstrate a greater surface-to-volume ratio. Statistical results reveal no significant bilateral asymmetries in either sex, although the effect sizes are larger in men, suggesting that women in this cohort show greater symmetry between the right and left muscles. The volumes measured in this imaging study consistently remain above the cadaver data, according to Van Eijden et al. (1997) and the MRI-based data reported by Heins et al. (2025). However, the values reported in this study were largely consistent with the median and mean values of recent MRI-based studies, such as Ng H. P. et al. (2009), or Lin et al. (2025) and are generally slightly below the reported mean volumes for women by Adisen et al. (2018). In comparison, published mean muscle lengths from the established literature are mostly comparable to the medians reported in this study (Van Eijden et al., 1997).

### 4.1 Sexual dimorphism

When examining the differences between male and female parameters in this study, it becomes clear that all reported volumes in men are higher than in women, highlighting the difficulty of comparing these results with data from mixed-sex cohorts in the literature. Regarding sex differences in masticatory muscle lengths, it is important to note that the average deviation of the evaluated muscle lengths between men and women is about 15%, whereas volume data show an approximately 40% sex difference. In mixed-sex cohorts, means and medians are less affected by uneven participant distributions than volume data. This may explain the greater consistency of the length data from Van Eijden et al. (1997) with this study, compared to the discrepancies in the volume data. The surface-to-volume ratio, as determined by the Rank-Biserial correlation, is higher in women than in men, reflecting thinner, less voluminous muscles, as shown by smaller anatomical cross-sectional areas. It is also important to recognize that nearly all male parameters, except for the surface-to-volume ratio, show medium-to-high differences relative to female data, as indicated by effect sizes from the Rank-Biserial test, although the right and left CSA values at 25% of the MMS length show no significant differences between males and females. This may be attributable to the small overall sample sizes and the unequal cohort sizes of seven males versus fifteen females. This interpretation is supported by the simulation-based *post hoc* power analysis, which showed the lowest power vales for the non-significant right and left CSA measurements at 25% of the MMS length (right: Power = 0.324; left: Power = 0.454), indication that these findings should be interpreted with caution rather than as evidence of no sex-related difference.

### 4.2 Bilateral symmetries and asymmetries

Statistical comparisons of the muscle sides between male and female data in this study reveal that, despite the absence of significant differences between right and left muscle sides, about one-third of the effect sizes for male parameters indicate large differences (*r* ≥ 0.5) between the right and left muscles. In contrast, the effect sizes for the female dataset fall within the small-to-medium range for muscle side differences (*r* < 0.5). Therefore, female values show better bilateral symmetry in muscle parameters, with a sample size (*n* = 15) more than twice as large as that of men (*n* = 7). This larger female sample size may provide more stable estimates of bilateral differences, whereas the larger effect sizes observed in smaller male cohort may represent overestimates. The potential operator dependency of the imaging-based landmark assessment was evaluated through repeated segmentation and measurement of four datasets by the same investigator. The predominantly good to excellent ICC values support the intra-observer reliability of the applied methods. However, the wide confidence intervals associated with several parameter-specific ICC estimates and the small number of repeated datasets limit the precision of the reliability assessment.

### 4.3 Comparison with the literature

In the methodology, Van Eijden et al. (1997) describe dissecting the masticatory muscles of three male and five female cadavers. The tendinous part and the contractile muscle volume were extracted and measured separately, which differs fundamentally from the reported methodology of Ng H. et al. (2009), Adisen et al. (2018), Lin et al. (2025) and this study. The percentages of tendon in muscles were reported according to Van Eijden et al. (1997) with 17.8% ± 3.3% for jaw-closing muscles (MM, MPM and MT) and 6.0% ± 1.9% for jaw-opening muscles (MPL), which may explain the substantial deviations between the volumes reported in the literature and the muscle volumes, including tendons, in this study. Additionally, the average age of the eight cadavers according to Van Eijden et al. (1997) was 70.5 years, which is substantially higher than the medians of the examined cohorts reported in this study and could be attributed to lower volumes due to the advanced age of the cadaver cohort (Weijs and Hillen, 1985; Ng H. P. et al., 2009). According to Van Eijden et al. (1997), the evaluated muscle lengths of the MMS, and MMP, as well as the MPM muscle show good agreement with the reported values in this study. In contrast, it is important to emphasize that the length of the MPL reported in the literature differs from the length value of this study. The length deviations could indicate increased operator dependency and consequently higher risk of error in the imaging-based landmark assessment method in this study compared to cadaver studies. Finally, the statistical data reported in the literature were derived from a mixed-sex cohort with a small sample size (Van Eijden et al., 1997), making it difficult to directly compare these values with the male and female data from this study.

Heins et al. (2025) investigated 41 head and neck oncology patients, including 18 women and 23 men, with a mean age of 64 years and an age range of 24–85 years, and reported muscle volumes as mixed-sex values. Bilateral average muscle volumes were derived using a validated deep learning segmentation algorithm with manual verification. The lower volumes reported in their study for the MMS and MMP, may therefore be explained by the higher mean age and the oncological disease status of the cohort, which could have contributed to reduced masticatory muscle volumes compared with the values reported in this study and other MRI-based studies.

The mean muscle volume values obtained by Ng H. et al. (2009) using a 1.5-T Siemens MR Scanner fall between the medians of the male and female values reported in this study. Due to the similar age range of the participants, according to Ng H. et al. (2009) with 20–25 years and the shape-based interpolation method, describing a semi-automatic segmentation process involving manual contouring of the muscles and program-based interpolation, it can be concluded that there is good descriptive agreement between the data reported from the literature and the data obtained using the “Grow from Seeds” method in this study. Furthermore, it is important to highlight that the muscle volume data reported by Ng H. et al. (2009) are based on a mixed cohort without specifying sex and a small sample size of twelve participants. The volume data according to Adisen et al. (2018), collected from twelve healthy women aged 20–27 years, demonstrate values for MT that are nearly identical to those reported in this study and slightly higher but still corresponding values for the MM and MPM. An important point to note is that the mean values reported by Adisen et al. (2018) are associated with a high standard deviation, indicating outliers or a skewed distribution, which may be explained by the small female sample size. The study by Lin et al. (2025) demonstrates volume measurements of the masticatory muscles that show good agreement with the MM, MPM and MPL volumes of the male cohort reported here, but they also reveal deviations from the female data. These discrepancies could be attributed to the unspecified sex distribution of the mixed cohort or to the different methodology used in this published literature. Lin et al. (2025) determined the muscle volumes using a diffusion MRI method that tracks the movements of water molecules in muscle fibers and thereby measures the functional expansion of the muscle fibers. It is important to emphasize that the diffusion spectrum imaging (DSI) method reported by Lin et al. (2025) has a low voxel resolution of 1.7 mm × 1.7 mm x 3 mm, along with a five-pixel tolerance for low-quality fibers in relation to the muscle boundaries, which, combined with the mixed-sex cohort, may explain the higher volumes observed in the masticatory muscles compared to this study.

### 4.4 Limitations

A major limitation of this study is the relatively small male sample size of seven participants, which reduces the statistical power of the male cohort. This limitation could therefore be crucial for the increased effect sizes observed in the muscle-side comparison within the male cohort and the two non-significant cross-sectional areas of the MMS after FDR correction. Furthermore the uneven sex distribution of the cohorts (men *n* = 7, women *n* = 15), limits the interpretation of bilateral asymmetries and sex differences. Although the simulation-based *post hoc* power analysis demonstrated sufficient power for the majority of sex-specific comparisons, several parameters showed limited power. Therefore, non-significant findings in these parameters should be interpreted cautiously.

#### 4.4.1 Challenges in comparative literature

The cohorts cited in the literature are often not analysed by sex, making it difficult to compare them with data reported in this study. The parameters from this research show that volumes are up to 40% higher in male than in female participants, limiting the use of mixed-sex cohorts for comparison. Consequently, volume parameters are substantially more influenced by sex than length parameters, making comparisons based on volume data prone to error and raising questions about the validity of using mixed-sex cohorts in biomechanical models to determine maximum forces.

#### 4.4.2 Methodological differences

There are notable differences in the methods used for data collection across the literature. More recent studies, such as Ng H. et al. (2009), Adisen et al. (2018), Lin et al. (2025), Heins et al. (2025), collect *in vivo* data using MR-imaging technology, whereas Van Eijden et al. (1997) collected data from cadavers. Additionally, the imaging methods segmented the muscle, including the muscle-tendon unit, because of the difficulty in identifying tendon portions in the digital MR slices, whereas Van Eijden et al. (1997) physically extracted the tendon part from the contractile muscle part. It is important to emphasize that the methodology described in the present study for segmenting the contact volumes between muscle and bone is operator-dependent and was performed exclusively by a single observer, which increases the potential for bias and measurement error in muscle parameters and makes an assessment of inter- or intra-observer reliability necessary. When comparing the landmark evaluation method used in this study to Merema et al. (2022), it is important to note that the thin, layer-like segmented contact volumes may be more prone to distortion of force application points, as contact volumes that are segmented too thickly or thinly can lead to misrepresentation of the center-of-mass. Another limitation of this study and the literature is that muscle atrophy occurs with advancing age (Ng H. P. et al., 2009), which leads to substantial differences when comparing studies of young adults (this study, Ng H. et al., 2009; Adisen et al., 2018; Lin et al., 2025) with studies with a mean age of 64 and 70.5 years according to Heins et al. (2025) and Van Eijden et al. (1997). A comparison of this study with other image-based literature shows a small sample size by Ng H. et al. (2009), with twelve participants and without specifying sex, which results in less precise muscle measurements. Adisen et al. (2018) applied a manual segmentation method to a small cohort of 12 female participants, which may explain the high standard deviation associated with increased operator dependence compared to semi-automatic methods. Lin et al. (2025) indicate a larger cohort of 25 healthy adult volunteers compared to other imaging research. However, they use a low-resolution approach, including a 5-pixel tolerance when determining track volumes with the DSI method, which could lead to volume overestimation at muscle boundaries.

## 5 Conclusion

The comprehensive, sex-specific dataset evaluated in this study provides a solid foundation for characterizing the masticatory muscles and offers broad support for future validation of biomechanical models. Our results demonstrate good consistency across diverse methodologies in the literature, confirming the effectiveness of semi-automatic segmentation on the imaging computing platform 3D Slicer and the developed center-of-mass-based segmentation method for identifying muscle landmarks to define biomechanical force vectors. The morphometric muscle parameters can thus be integrated into general sex-specific muscle force models and used to simulate masticatory movements, stress analyses, and treatment planning. However, the relatively small sample size and uneven sex distribution should be considered when interpreting the statistical robustness and generalizability of the findings.

## Data Availability

The raw data supporting the conclusions of this article will be made available by the authors, without undue reservation.
