# Supplementary material for: MRI-based anatomical and biomechanical characterization of the human masticatory muscles
Source: Front Bioeng Biotechnol. 2026 Sep 14;14:1888163. doi: 10.3389/fbioe.2026.1888163 (PMC13617056; doi:10.3389/fbioe.2026.1888163)
Supplement: Supplementary file 1 [file DataSheet1.pdf]

Supplementary Table 1.
Statistical analysis of male masticatory muscle parameters for the right and left sides.

			RIGHT						LEFT					
**Muscle**	**Parameter**	***n***	***Q1***	**Median**	***Q3***	**IQR**	**Min**	**Max**	***Q1***	**Median**	***Q3***	**IQR**	**Min**	**Max**
**M. Masseter**	Volume	7	31.65	34.99	37.79	6.14	28.47	48.02	30.02	33.94	37.81	7.79	27.51	46.56
**M. Masseter Superficialis**	Length		45.32	46.38	47.71	2.39	41.56	48.97	44.95	45.63	47.44	2.49	41.08	48.82
	CSA 25%		3.14	3.41	3.88	0.74	2.12	5.25	3.20	3.57	3.65	0.45	2.27	3.96
	CSA 50%		4.83	5.34	5.87	1.03	4.08	7.33	4.68	5.11	5.57	0.89	3.57	6.62
	CSA 75%		6.04	6.68	7.18	1.14	5.26	8.30	5.84	6.59	7.31	1.47	4.74	7.70
	Volume		23.91	26.47	29.30	5.39	21.28	37.71	23.36	25.68	28.94	5.58	21.45	36.66
	Surface		55.91	60.69	62.69	6.78	51.21	71.23	53.85	58.70	63.69	9.84	50.17	71.15
	Surface/Volume Ratio		2.06	2.25	2.39	0.33	1.89	2.55	2.07	2.29	2.40	0.33	1.94	2.45
**M. Masseter Profunda**	Length		22.65	25.37	26.61	3.96	20.86	29.59	22.80	25.02	26.36	3.56	21.34	29.95
	CSA 25%		2.75	3.08	3.96	1.22	2.55	4.22	2.87	3.05	3.52	0.65	2.19	4.16
	CSA 50%		2.88	3.26	4.05	1.16	2.73	4.36	3.08	3.56	3.88	0.80	2.42	4.49
	CSA 75%		2.11	2.39	3.22	1.10	1.90	3.48	2.24	2.65	2.82	0.58	2.01	3.13
	Volume		7.58	8.52	9.13	1.55	6.22	10.31	6.65	7.90	9.04	2.39	6.06	9.90
	Surface		27.69	28.15	28.91	1.22	24.14	30.78	24.39	25.61	27.45	3.06	21.68	32.96
	Surface/Volume Ratio		3.15	3.27	3.70	0.55	2.98	3.88	3.18	3.31	3.46	0.28	2.95	4.08
**M. Pterygoideus Medialis**	Length		39.25	40.78	46.15	6.90	37.65	49.25	38.51	40.53	45.93	7.43	36.27	48.44
	CSA 25%		1.53	1.96	1.97	0.44	1.37	2.00	1.72	1.79	1.97	0.24	1.68	2.13
	CSA 50%		2.79	2.97	3.12	0.33	2.76	3.37	2.79	3.07	3.13	0.34	2.69	3.18
	CSA 75%		3.37	3.80	4.19	0.82	2.90	4.70	3.25	3.61	3.88	0.63	2.70	4.09
	Volume		12.29	13.76	13.92	1.63	9.88	18.29	11.10	12.64	13.63	2.54	9.45	17.44
	Surface		33.98	36.88	39.33	5.34	30.35	45.43	32.48	33.49	38.91	6.43	30.13	45.41
	Surface/Volume Ratio		2.63	2.84	2.90	0.27	2.48	3.07	2.71	2.92	2.94	0.23	2.60	3.19
**M. Pterygoideus Lateralis**	Length		29.52	30.00	31.12	1.60	28.27	32.28	29.04	29.46	30.61	1.57	27.71	31.22
	CSA 25%		3.09	3.24	3.57	0.48	2.87	3.66	3.33	3.39	3.56	0.23	3.12	3.77
	CSA 50%		3.76	3.90	3.93	0.17	3.67	4.68	3.72	3.90	4.06	0.35	3.47	4.85
	CSA 75%		3.43	3.88	4.12	0.69	3.28	4.26	3.50	4.00	4.04	0.54	3.36	4.07
	Volume		10.20	10.50	11.47	1.27	8.89	12.36	10.08	10.35	11.71	1.63	8.99	11.93
	Surface		26.47	27.02	29.20	2.74	25.21	30.01	26.48	26.96	29.17	2.69	25.34	30.31
	Surface/Volume Ratio		2.52	2.55	2.63	0.11	2.43	2.84	2.51	2.54	2.67	0.16	2.49	2.82
**M. Temporalis Anterior**	Length		68.35	73.00	76.59	8.24	64.27	76.70	68.83	72.44	75.69	6.85	64.50	76.74
**M. Temporalis Medialis**	Length		94.56	97.62	99.07	4.51	89.35	104.30	94.57	95.69	100.12	5.55	88.80	104.20
**M. Temporalis Posterior**	Length		108.45	109.40	110.70	2.25	108.00	113.70	108.95	109.90	111.30	2.35	107.60	113.50
**M. Temporalis**	Volume		59.82	62.80	68.18	8.35	48.91	83.21	59.69	63.50	64.19	4.49	47.14	70.65
	Surface		197.00	220.33	234.14	37.14	176.83	238.32	201.56	214.45	217.71	16.15	190.77	251.30
	Surface/Volume Ratio		3.29	3.34	3.49	0.20	2.78	3.80	3.28	3.32	3.72	0.44	3.11	4.20

Abbreviations: number of participants (*n*); cross-sectional area (CSA)
