# Supplementary material for: MRI-based anatomical and biomechanical characterization of the human masticatory muscles
Source: Front Bioeng Biotechnol. 2026 Sep 14;14:1888163. doi: 10.3389/fbioe.2026.1888163 (PMC13617056; doi:10.3389/fbioe.2026.1888163)
Supplement: Supplementary file 2 [file DataSheet2.pdf]

Supplementary Table 2.
Statistical analysis of female masticatory muscle parameters for the right and left sides.

			RIGHT						LEFT					
**Muscle**	**Parameter**	***n***	***Q1***	**Median**	***Q3***	**IQR**	**Min**	**Max**	***Q1***	**Median**	***Q3***	**IQR**	**Min**	**Max**
**M. Masseter**	Volume	15	23.09	24.44	24.98	1.89	17.55	32.18	22.44	23.63	26.84	4.41	17.79	33.44
**M. Masseter Superficialis**	Length		38.23	40.72	42.21	3.98	36.08	45.32	39.64	41.45	42.75	3.11	36.22	44.07
	CSA 25%		2.50	3.18	3.39	0.89	1.92	3.69	2.50	2.95	3.12	0.62	1.86	4.02
	CSA 50%		4.02	4.40	4.54	0.52	2.98	5.11	3.82	3.98	4.67	0.86	2.88	5.30
	CSA 75%		4.73	4.96	5.37	0.64	3.58	6.16	4.41	4.89	5.65	1.24	3.00	6.76
	Volume		18.10	18.73	19.38	1.28	13.01	25.40	16.78	17.37	20.70	3.92	13.46	27.41
	Surface		46.23	46.71	48.39	2.15	39.56	57.77	44.13	47.19	50.48	6.35	40.85	57.99
	Surface/Volume Ratio		2.45	2.48	2.61	0.16	2.27	3.15	2.37	2.55	2.69	0.32	2.12	3.22
**M. Masseter Profunda**	Length		19.44	19.88	21.33	1.89	18.60	27.52	19.53	20.84	21.49	1.96	19.30	26.37
	CSA 25%		2.31	2.47	2.79	0.48	1.79	3.28	2.30	2.51	2.79	0.49	1.64	3.17
	CSA 50%		2.35	2.60	2.80	0.45	1.77	3.19	2.30	2.51	2.81	0.51	2.04	3.21
	CSA 75%		1.84	2.01	2.18	0.34	1.52	2.54	1.83	1.94	2.45	0.62	1.69	2.74
	Volume		5.00	5.27	5.78	0.78	4.36	7.16	5.36	5.81	6.15	0.79	4.33	6.64
	Surface		20.03	20.46	22.63	2.60	17.50	25.05	21.26	21.95	22.66	1.40	17.96	25.06
	Surface/Volume Ratio		3.69	3.86	3.97	0.27	3.50	4.53	3.60	3.75	3.92	0.32	3.46	4.21
**M. Pterygoideus Medialis**	Length		36.37	37.00	38.03	1.66	31.98	40.46	34.51	36.38	37.87	3.36	31.94	39.83
	CSA 25%		1.13	1.26	1.47	0.35	1.05	1.91	1.21	1.40	1.58	0.38	0.96	1.82
	CSA 50%		1.87	2.03	2.49	0.62	1.37	2.99	1.92	2.31	2.47	0.55	1.57	3.22
	CSA 75%		2.28	2.52	3.01	0.74	1.85	3.47	2.44	2.71	2.92	0.49	2.08	3.29
	Volume		7.50	7.66	8.92	1.43	6.93	12.67	7.72	8.52	9.86	2.14	6.34	12.16
	Surface		24.55	25.84	28.66	4.12	23.19	35.42	25.51	27.21	29.59	4.08	22.57	34.24
	Surface/Volume Ratio		3.07	3.23	3.41	0.33	2.71	3.52	2.99	3.10	3.22	0.23	2.82	3.59
**M. Pterygoideus Lateralis**	Length		25.22	27.25	29.54	4.32	21.31	31.88	25.58	26.49	28.86	3.29	24.15	32.15
	CSA 25%		2.61	2.81	2.92	0.31	2.25	3.14	2.60	2.77	2.83	0.23	2.34	3.24
	CSA 50%		2.75	3.09	3.25	0.50	2.55	3.61	2.81	3.00	3.15	0.34	2.49	3.54
	CSA 75%		2.35	2.65	2.89	0.54	2.18	3.22	2.47	2.58	2.72	0.25	1.81	3.24
	Volume		6.62	6.94	7.78	1.16	6.25	9.64	6.33	7.04	7.44	1.11	6.06	9.39
	Surface		20.43	21.28	22.94	2.50	18.38	26.54	19.95	20.74	22.35	2.40	18.31	25.93
	Surface/Volume Ratio		2.91	2.99	3.10	0.20	2.64	3.21	2.94	3.00	3.03	0.09	2.62	3.33
**M. Temporalis Anterior**	Length		62.23	65.03	70.07	7.83	58.68	76.08	62.42	65.11	70.06	7.64	58.75	76.38
**M. Temporalis Medialis**	Length		86.80	89.83	95.48	8.68	80.04	97.84	86.50	88.57	94.71	8.22	80.25	96.00
**M. Temporalis Posterior**	Length		99.44	103.10	105.95	6.52	95.12	115.40	99.57	103.40	106.15	6.59	93.26	115.70
**M. Temporalis**	Volume		43.89	46.80	49.39	5.50	38.81	62.36	41.85	47.03	50.88	9.03	37.72	59.48
	Surface		174.64	185.46	193.72	19.09	162.76	206.80	173.38	187.15	195.65	22.27	164.93	214.92
	Surface/Volume Ratio		3.56	3.90	4.13	0.57	3.02	4.66	3.61	3.99	4.31	0.69	3.25	4.69

Abbreviations: number of participants (*n*); cross-sectional area (CSA)
