# Supplementary material for: MRI-based anatomical and biomechanical characterization of the human masticatory muscles
Source: Front Bioeng Biotechnol. 2026 Sep 14;14:1888163. doi: 10.3389/fbioe.2026.1888163 (PMC13617056; doi:10.3389/fbioe.2026.1888163)
Supplement: Supplementary file 3 [file DataSheet3.pdf]

Supplementary Table 3.
Statistical analysis of male masticatory muscle parameters.

Muscle	Parameter	*n*	*Q1*	Median	*Q3*	IQR	MIN	MAX
**M. Masseter Superficialis**	Length	7	44.94	46.12	48.06	3.12	41.08	48.97
	CSA 25%		3.11	3.49	3.70	0.58	2.12	5.25
	CSA 50%		4.61	5.27	5.67	1.07	3.57	7.33
	CSA 75%		5.86	6.63	7.44	1.58	4.74	8.30
	Volume		23.44	26.08	29.55	6.12	21.28	37.71
	Surface		54.42	59.70	63.69	9.27	50.17	71.23
	Surface/Volume Ratio		2.06	2.27	2.41	0.35	1.89	2.55
**M. Masseter Profunda**	Length		22.23	25.20	26.43	4.20	20.86	29.95
	CSA 25%		2.82	3.06	3.85	1.04	2.19	4.22
	CSA 50%		2.81	3.51	4.00	1.19	2.42	4.49
	CSA 75%		2.08	2.52	3.02	0.94	1.70	3.48
	Volume		6.87	8.12	9.20	2.33	6.06	10.31
	Surface		24.94	27.69	28.98	4.05	21.68	32.96
	Surface/Volume Ratio		3.15	3.29	3.55	0.41	2.95	4.08
**M. Pterygoideus Medialis**	Length		38.64	40.66	46.29	7.65	36.27	49.25
	CSA 25%		1.69	1.85	1.97	0.28	1.37	2.13
	CSA 50%		2.78	3.01	3.15	0.36	2.69	3.37
	CSA 75%		3.17	3.74	4.03	0.85	2.70	4.70
	Volume		11.25	13.34	13.80	2.54	9.45	18.29
	Surface		32.54	36.18	39.60	7.06	30.13	45.43
	Surface/Volume Ratio		2.66	2.85	2.94	0.28	2.48	3.19
**M. Pterygoideus Lateralis**	Length		29.09	29.98	30.84	1.75	27.71	32.28
	CSA 25%		3.19	3.37	3.59	0.40	2.87	3.77
	CSA 50%		3.71	3.90	3.94	0.22	3.47	4.85
	CSA 75%		3.43	3.94	4.06	0.63	3.28	4.26
	Volume		10.10	10.43	11.62	1.52	8.89	12.36
	Surface		26.31	27.00	29.40	3.09	25.21	30.31
	Surface/Volume Ratio		2.51	2.55	2.67	0.16	2.43	2.84
**M. Temporalis Anterior**	Length		68.38	72.72	76.35	7.96	64.27	76.74
**M. Temporalis Medialis**	Length		94.30	96.80	99.57	5.27	88.80	104.30
**M. Temporalis Posterior**	Length		108.63	109.45	111.55	2.93	107.60	113.70
**M. Temporalis**	Volume		59.65	63.15	65.64	5.99	47.14	83.21
	Surface		197.91	215.12	228.31	30.40	176.83	251.30
	Surface/Volume Ratio		3.27	3.34	3.56	0.29	2.78	4.20

Abbreviations: number of participants (*n*); cross-sectional area (CSA)
