# Supplementary material for: MRI-based anatomical and biomechanical characterization of the human masticatory muscles
Source: Front Bioeng Biotechnol. 2026 Sep 14;14:1888163. doi: 10.3389/fbioe.2026.1888163 (PMC13617056; doi:10.3389/fbioe.2026.1888163)
Supplement: Supplementary file 4 [file DataSheet4.pdf]

Supplementary Table 4.
Statistical analysis of female masticatory muscle parameters.

Muscle	Parameter	*n*	*Q1*	Median	*Q3*	IQR	MIN	MAX
**M. Masseter Superficialis**	Length	15	38.61	40.92	42.23	3.62	36.08	45.32
	CSA 25%		2.49	2.97	3.32	0.83	1.86	4.02
	CSA 50%		3.91	4.29	4.61	0.70	2.88	5.30
	CSA 75%		4.65	4.93	5.56	0.91	3.00	6.76
	Volume		16.99	18.68	20.44	3.45	13.01	27.41
	Surface		45.15	46.95	48.74	3.59	39.56	57.99
	Surface/Volume Ratio		2.44	2.54	2.66	0.22	2.12	3.22
**M. Masseter Profunda**	Length		19.50	20.79	21.34	1.84	18.60	27.52
	CSA 25%		2.30	2.49	2.80	0.50	1.64	3.28
	CSA 50%		2.33	2.56	2.80	0.47	1.77	3.21
	CSA 75%		1.81	2.01	2.31	0.50	1.52	2.74
	Volume		5.16	5.52	6.08	0.92	4.33	7.16
	Surface		20.34	21.37	22.66	2.32	17.50	25.06
	Surface/Volume Ratio		3.61	3.84	3.94	0.33	3.45	4.52
**M. Pterygoideus Medialis**	Length		35.03	36.82	37.89	2.86	31.94	40.46
	CSA 25%		1.14	1.31	1.55	0.41	0.96	1.91
	CSA 50%		1.88	2.13	2.50	0.62	1.37	3.22
	CSA 75%		2.35	2.58	2.93	0.58	1.85	3.47
	Volume		7.53	8.17	9.25	1.72	6.34	12.67
	Surface		24.81	26.27	29.03	4.22	22.57	35.14
	Surface/Volume Ratio		3.02	3.19	3.40	0.38	2.71	3.59
**M. Pterygoideus Lateralis**	Length		25.48	27.14	28.95	3.47	21.31	32.15
	CSA 25%		2.60	2.77	2.89	0.28	2.25	3.24
	CSA 50%		2.76	3.01	3.21	0.45	2.49	3.61
	CSA 75%		2.41	2.60	2.85	0.44	1.81	3.24
	Volume		6.52	6.99	7.45	0.93	6.06	9.64
	Surface		20.16	21.05	22.41	2.25	18.31	26.54
	Surface/Volume Ratio		2.93	2.99	3.05	0.13	2.62	3.33
**M. Temporalis Anterior**	Length		62.26	65.07	70.65	8.39	58.68	76.38
**M. Temporalis Medialis**	Length		86.43	89.22	95.32	8.89	80.04	97.84
**M. Temporalis Posterior**	Length		99.45	103.25	105.98	6.53	93.26	115.70
**M. Temporalis**	Volume		43.10	46.92	50.47	7.38	37.72	62.36
	Surface		174.12	186.31	197.07	22.94	162.76	214.92
	Surface/Volume Ratio		3.57	3.96	4.27	0.70	3.02	4.69

Abbreviations: number of participants (*n*); cross-sectional area (CSA)
