# Supplementary material for: MRI-based anatomical and biomechanical characterization of the human masticatory muscles
Source: Front Bioeng Biotechnol. 2026 Sep 14;14:1888163. doi: 10.3389/fbioe.2026.1888163 (PMC13617056; doi:10.3389/fbioe.2026.1888163)
Supplement: Supplementary file 5 [file DataSheet5.pdf]

Supplementary Table 5.
Muscle volume and length comparison of the M. masseter with previously published reference values from the literature.

Literature	Sex	Muscle	Parameter	Unit	*n*	*Q1*	Median	*Q3*	IQR	Min	Max	Mean	SD
this study	M	MMR	Muscle Volume	cm³	7	31.65	34.99	37.79	6.14	28.47	48.02		
this study	F	MMR	Muscle Volume	cm³	15	23.09	24.44	24.98	1.89	17.55	32.18		
**Ng et al. 2009**	M/F	MMR	Muscle Volume	cm³	12							29.65	6.77
this study	M	MML	Muscle Volume	cm³	7	30.02	33.94	37.81	7.79	27.51	46.56		
this study	F	MML	Muscle Volume	cm³	15	22.44	23.63	26.84	4.41	17.79	33.44		
**Ng et al. 2009**	M/F	MML	Muscle Volume	cm³	12							29.54	5.19
**Lin et al. 2025**	M/F	MM	Muscle Volume (DSI)	cm³	25							36.40	8.06
this study	F	MM	Muscle Volume (total)	cm³	15	45.67	47.49	51.67	6.00	35.34	65.62		
**Adisen et al. 2018**	F	MM	Muscle Volume (total)	cm³	12							54.90	13.40
this study	M	MMSR	Muscle Volume	cm³	7	23.91	26.47	29.30	5.39	21.28	37.71		
this study	M	MMSL	Muscle Volume	cm³	7	23.36	25.68	28.94	5.58	21.45	36.66		
this study	F	MMSR	Muscle Volume	cm³	15	18.10	18.73	19.38	1.28	13.01	25.40		
this study	F	MMSL	Muscle Volume	cm³	15	16.78	17.37	20.70	3.92	13.46	27.41		
**Eijden et al. 1997**	M/F	MMS	Muscle Volume (contractile)	cm³	8							14.79	3.98
**Heins et al. 2025**	M/F	MMS	Muscle Volume	cm³	41							14.30	3.80
this study	M	MMPR	Muscle Volume	cm³	7	7.58	8.52	9.13	1.55	6.22	10.31		
this study	M	MMPL	Muscle Volume	cm³	7	6.65	7.90	9.04	2.39	6.06	9.90		
this study	F	MMPR	Muscle Volume	cm³	15	5.00	5.27	5.78	0.78	4.36	7.16		
this study	F	MMPL	Muscle Volume	cm³	15	5.36	5.81	6.15	0.79	4.33	6.64		
**Eijden et al. 1997**	M/F	MMP	Muscle Volume (contractile)	cm³	8							5.36	1.20
**Heins et al. 2025**	M/F	MMP	Muscle Volume	cm³	41							3.80	1.0
this study	M	MMS	Muscle Length	mm	7	44.94	46.12	48.06	3.12	41.08	48.97		
this study	F	MMS	Muscle Length	mm	15	38.61	40.92	42.23	3.62	36.08	45.32		
**Eijden et al. 1997**	M/F	MMS	Muscle Length	mm	8							45.60	4.20
this study	M	MMP	Muscle Length	mm	7	22.23	25.20	26.43	4.20	20.86	29.95		
this study	F	MMP	Muscle Length	mm	15	19.50	20.79	21.34	1.84	18.60	27.52		
**Eijden et al. 1997**	M/F	MMP	Muscle Length	mm	8							25.30	3.90

Abbreviations. male (M); female (F); Male/Female (M/F); M. masseter right (MMR); M. masseter left (MML); M. masseter (MM); M. masseter superficialis right (MMSR); M. masseter superficialis left (MMSL); M. masseter superficialis (MMS); M. masseter profunda right (MMPR); M. masseter pro-funda left (MMPL); M. masseter profunda (MMP); number of participants (*n*); diffusion tensor imaging (DSI); muscle volume without tendon (contractile); sum of right and left muscle side (total)
