# Supplementary material for: MRI-based anatomical and biomechanical characterization of the human masticatory muscles
Source: Front Bioeng Biotechnol. 2026 Sep 14;14:1888163. doi: 10.3389/fbioe.2026.1888163 (PMC13617056; doi:10.3389/fbioe.2026.1888163)
Supplement: Supplementary file 6 [file DataSheet6.pdf]

Supplementary Table 6. Muscle volume and length comparison of the M. pterygoideus with previously published reference values from the literature.

Literature	Sex	Muscle	Parameter	Unit	*n*	*Q1*	Median	*Q3*	IQR	Min	Max	Mean	SD
this study	M	PMR	Muscle Volume	cm³	7	12.29	13.76	13.92	1.63	9.88	18.29		
this study	F	PMR	Muscle Volume	cm³	15	7.50	7.66	8.92	1.43	6.93	12.67		
**Ng et al. 2009**	M/F	PMR	Muscle Volume	cm³	12							8.92	1.54
this study	M	PML	Muscle Volume	cm³	7	11.10	12.64	13.63	2.54	9.45	17.44		
this study	F	PML	Muscle Volume	cm³	15	7.72	8.52	9.86	2.14	6.34	12.16		
**Ng et al. 2009**	M/F	PML	Muscle Volume	cm³	12							8.69	1.66
**Lin et al. 2025**	M/F	PM	Muscle Volume (DSI)	cm³	25	11.09	12.19	13.93	2.84				
**Eijden et al. 1997**	M/F	PM	Muscle Volume (contractile)	cm³	8							7.73	2.12
**Heins et al. 2025**	M/F	PM	Muscle Volume	cm³	41							8.20	2.00
this study	F	PM	Muscle Volume (total)	cm³	15	15.13	16.33	18.43	3.30	13.27	24.83		
**Adisen et al. 2018**	F	PM	Muscle Volume (total)	cm³	12							18.50	6.70
this study	M	PLR	Muscle Volume	cm³	7	10.20	10.50	11.47	1.27	8.89	12.36		
this study	F	PLR	Muscle Volume	cm³	15	6.62	6.94	7.78	1.16	6.25	9.64		
**Ng et al. 2009**	M/F	PLR	Muscle Volume	cm³	12							10.23	1.76
this study	M	PLL	Muscle Volume	cm³	7	10.08	10.35	11.71	1.63	8.99	11.93		
this study	F	PLL	Muscle Volume	cm³	15	6.33	7.04	7.44	1.11	6.06	9.39		
**Ng et al. 2009**	M/F	PLL	Muscle Volume	cm³	12							9.47	2.15
**Lin et al. 2025**	M/F	PL	Muscle Volume (DSI)	cm³	25							10.55	2.16
**Eijden et al. 1997**	M/F	PL	Muscle Volume (contractile)	cm³	8							8.49	1.75
this study	F	PL	Muscle Volume (total)	cm³	15	12.89	14.08	14.88	1.98	12.46	19.04		
**Adisen et al. 2018**	F	PL	Muscle Volume (total)	cm³	12							19.30	4.10
this study	M	PM	Muscle Length	mm	7	38.64	40.66	46.29	7.65	36.27	49.25		
this study	F	PM	Muscle Length	mm	15	35.03	36.82	37.89	2.86	31.94	40.46		
**Eijden et al. 1997**	M/F	PM A	Muscle Length	mm	8							37.90	6.00
**Eijden et al. 1997**	M/F	PM P	Muscle Length	mm	8							44.40	5.50
this study	M	PL	Muscle Length	mm	7	29.09	29.98	30.84	1.75	27.71	32.28		
this study	F	PL	Muscle Length	mm	15	25.48	27.14	28.95	3.47	21.31	32.15		
**Eijden et al. 1997**	M/F	PL I	Muscle Length	mm	8							32.60	3.10
**Eijden et al. 1997**	M/F	PL S	Muscle Length	mm	8							31.30	2.90

Abbreviations. male (M); female (F); Male/Female (M/F); M. pterygoideus medialis right (PMR); M. pterygoideus medialis left (PML); M. pterygoideus medialis (PM); M. pterygoideus lateralis right (PLR); M. pterygoideus lateralis left (PLL); M. pterygoideus lateralis (PL); PM, M. pterygoideus medialis; M. pterygoideus medialis anterior (PM A); M. pterygoideus medialis posterior (PM P); M. pterygoideus lateralis inferior (PL I); M. pterygoideus lateralis superior (PL S); number of participants (*n*); diffusion tensor imaging (DSI); muscle volume without tendon (contractile); sum of right and left muscle side (total)
