# Supplementary material for: MRI-based anatomical and biomechanical characterization of the human masticatory muscles
Source: Front Bioeng Biotechnol. 2026 Sep 14;14:1888163. doi: 10.3389/fbioe.2026.1888163 (PMC13617056; doi:10.3389/fbioe.2026.1888163)
Supplement: Supplementary file 7 [file DataSheet7.pdf]

Supplementary Table 7. Muscle volume comparison of the M. temporalis with previously published reference values from the literature.

Literature	Sex	Muscle	Parameter	Unit	*n*	*Q1*	Median	*Q3*	IQR	Min	Max	Mean	SD
this study	M	TR	Muscle Volume	cm³	7	59.82	62.80	68.18	8.35	48.91	83.21		
this study	F	TR	Muscle Volume	cm³	15	43.89	46.80	49.39	5.50	38.81	62.36		
**Ng et al. 2009**	M/F	TR	Muscle Volume	cm³	12							48.80	8.48
this study	M	TL	Muscle Volume	cm³	7	59.69	63.50	64.19	4.49	47.14	70.65		
this study	F	TL	Muscle Volume	cm³	15	41.85	47.03	50.88	9.03	37.72	59.48		
**Ng et al. 2009**	M/F	TL	Muscle Volume	cm³	12							47.70	7.37
**Eijden et al. 1997**	M/F	T	Muscle Volume (contractile)	cm³	8							34.79	8.27
this study	F	T	Muscle Volume (total)	cm³	15	86.23	93.83	98.42	12.19	76.53	120.91		
**Adisen et al. 2018**	F	T	Muscle Volume (total)	cm³	12							92.40	12.10

Abbreviations: M male (M); female (F); Male/Female (M/F); M. temporalis right (TR); M. temporalis left (TL); M. temporalis (T); number of participants (*n*); diffusion tensor imaging (DSI); muscle volume without tendon (contractile); sum of right and left muscle side (total)
